# Effect of n-3 fatty acids on the expression of inflammatory genes in THP-1 macrophages

**DOI:** 10.1186/s12944-016-0241-4

**Published:** 2016-04-05

**Authors:** Bénédicte Allam-Ndoul, Frédéric Guénard, Olivier Barbier, Marie-Claude Vohl

**Affiliations:** Institute of Nutrition and Functional Foods (INAF), Laval University, Pavillon des Services, 2440 Hochelaga Blvd, Québec, Québec G1V 0A6 Canada; Laboratory of Molecular Pharmacology, CHU de Quebec Research Center, 2705, boulevard Laurier R-4720, Québec, Québec G1V 4G2 Canada

**Keywords:** Omega-3, Eicosapentaenoic acid, Docosahexaenoic acid, Inflammation, THP-1, Genes

## Abstract

**Background:**

Uncontrolled inflammation participates in the development of inflammatory diseases. Beneficial effects of polyunsaturated fatty acids belonging to the n-3 family such as eicosapentaenoic acid (EPA) and docosahexaenoic acid (DHA) on inflammation have been reported.

The present study investigates the basal effects of EPA, DHA and a mixture EPA + DHA on the expression of 10 genes (*AKT1, MAPK, NFKB, TNFA, IL1Β, MCP1, ALOX5, PTGS2, MGST1*and *NOS2*) related to inflammation in unstimulated cultured THP1 macrophages. Cells were incubated for 24 h with n-3 PUFAs (50 μM and 10 μM EPA, DHA, EPA + DHA). Expression levels of inflammatory genes were analyzed by real-time PCR.

**Results:**

50 μM, 10 μM EPA and 50 μM EPA + DHA decreased the expression of genes involved in the NF-κB pathway (*MAPK*, *AKT1*, and *NFKB*). Treatment with 50 μM, 10 μM EPA, 50 μM DHA and EPA + DHA decreased expression levels of cytokines genes *IL1Β* and *MCP1. TNFA* expression was decreased by 50 μM, 10 μM of EPA, DHA and with 50 μM EPA + DHA. Two genes involved in the fatty acid metabolism (*PTGS2* and *ALOX5*) were also modulated by the n-3 FAs. 50 μM of DHA and EPA + DHA inhibited *PTGS2* expression when the two concentrations of EPA, 50 μM DHA and EPA + DHA inhibited *ALOX5* expression. Finally, the effects of n-3 FAs were studied among genes involved in the oxidative stress. 50 μM of each fatty acid increased *MGST1* expression. Both concentration of EPA and 50 μM DHA decreased *NOS2* expression.

**Conclusion:**

EPA seems to be more effective than DHA and EPA + DHA in modulating expression levels of selected inflammatory genes. The concentration of 50 μM was globally more effective than 10 μM.

## Background

Inflammation is part of the body’s immediate response to injury. It starts the immunologic elimination of invading pathogens and toxins in order to repair damaged tissues. During this stage, immune cells produce inflammatory molecules participating in the destruction of foreign agents [6, 31]. Although inflammation is a normal response, it can harm host tissues when uncontrolled. Such inappropriate inflammatory responses are in part characterized by an abnormal production of inflammatory mediators by peripheral blood mononuclear cells (PBMCs) triggering inflammatory diseases [8, 20, 32].

Polyunsaturated fatty acids (PUFAs) are natural constituents of the diet, having a wide spectrum of physiological roles [5, 8, 28]. Omega-6 (n-6) and omega-3 (n-3) fatty acids (FAs) are two classes of PUFAs. Linoleic acid (n-6 fatty acids) and alpha linolenic acid ((ALA) n-3 FAs) are essential FAs. They cannot be synthetized by the body and must be provided through the diet. The major end product of n-6 FAs is arachidonic acid (AA). AA generally leads to the production of pro-inflammatory eicosanoids. ALA is the precursor of eicosapentaenoic (EPA, 20:5n-3) and docosahexaenoic (DHA, 22:6n-3) acids producing anti-inflammatory eicosanoids involved in the resolution of inflammation [4, 29]. Unfortunately, cellular mechanisms by which n-3 FAs act on immune cells are not completely understood.

In vitro studies revealed that both EPA and DHA may influence the functional responses of immune cells to stimulation by reducing the production of pro-inflammatory mediators. Early studies in experimental models of autoimmunity [15] and clinical trials on fish oil in patients with rheumatoid arthritis [13] demonstrated significant anti-inflammatory activity of the combination of EPA and DHA. The beneficial effects of n-3 FAs are thought to result from their anti-inflammatory effects, in part through changes in gene expression levels. In women with type 2 diabetes and without hypertiglyceridemia Kabir et al. showed that expression levels of inflammation-related genes were reduced in adipose tissue after n-3 FA supplementation [10]. In the same manner, a transcriptomic study in elderly subjects by Bowens et al. showed that a supplementation with 1.8 g of EPA + DHA per day could alter gene expression toward a more anti-inflammatory profile among participants without inflammatory disease [3]. Finally, our team demonstrated that n-3 FAs supplementation (1.9 g EPA+ 1.1 g DHA/day during 6 weeks) in healthy men and women could alter gene expression levels of inflammation-related genes [24]. A decrease of the expression of *TNFA* (37 %) and *MGST1* (17 %) was seen following this supplementation, reinforcing the notion that EPA and DHA have an anti-inflammatory effect. This study also showed an alteration of pathways biologically related to inflammation and influenced by n-3 FAs supplementation. In fact, NFKB and oxidative stress signalling pathways were modulated after this supplementation [24].

It is important to further understand the molecular mechanisms underlying beneficial effects of PUFAs, such as EPA and DHA, in non-inflamed human immune cells. Therefore, the aim of this study was to investigate the effect of EPA, DHA and a mixture of EPA and DHA on expression levels of genes involved in inflammation in THP-1 macrophages were the inflammation was not induced. This study was realized on THP-1 macrophages because of the similarity of their transcriptomic profile with human primary cells [22].

## Methods

### Reagents and cell lines

Phosphate-buffered saline (PBS) solution was obtained from Life Technologies (Burlington, Canada). Cell culture media, Roswell Park Memorial Institute medium 1640 (RPMI), Fetal Bovine Serum (FBS), penicillin and streptomycin media supplements, phorbol 12-myristate 13-acetate (PMA), dimethyl sulfoxide (DMSO) were purchased from Thermo Scientific (Walthman, USA). EPA, DHA and reagents for reverse transcription were obtained from Applied biosystems (Oakville, Canada).

### Cell culture and Fatty acids treatment

The human THP-1 cell line, an acute monocytic leukemia cell line (American Type Culture Collection (ATCC), Rockville, MD, USA), was cultured in RPMI-1640 media supplemented with 10 % FBS, penicillin (100 U/ml) and streptomycin (100 μM/ml) at 37 °C in a 5 % CO_2_ incubator. To induce differentiation of monocytes into macrophages, 10^6^ cells per ml were seeded into 12 well plates, with 200 nM of PMA for 72 h. After incubation, non-attached cells were removed by aspiration, and the adherent cells were washed with PBS three times. Preliminary tests were done to determine the optimum conditions for this study which were an incubation of 24 h in 50 and 10 μM of n-3 FAs. Stock solutions of FAs (EPA-DMSO 330 nM and DHA-DMSO 760nM) prepared in serum-free RPMI 1640 medium were diluted in culture medium to obtain 50 and 10 μM concentrations. Cells were thereafter incubated in 50 and 10 μM of EPA, DHA and in 50 μM (25 μM EPA + 25 μM DHA) or 10 μM (5 μM EPA + 5 μM DHA) EPA + DHA for 24 h. The controls in this experiment were THP-1 cells incubated with the vehicle, DMSO.

### RNA isolation and quantitative real-time PCR

After 24 h, total RNA was extracted using RNeasy Mini Kit (Qiagen) following the protocol provided. Then cDNA was produced from RNA using High Capacity Transcription Kit (Applied biosystems). Real-time PCR was used to determine the expression of several inflammatory genes (Table 1) related to FA metabolism, FA receptors, oxidative stress, *NF-κB* signalling or toll-like receptor signaling. PCR samples were normalized against 18S gene expression. Primers and TaqMan® probes were obtained from Applied Biosystems (Table 1). Finally, each experimental treatment was performed three times and replicates were analyzed.

**Table 1:** Genes and primers sets for real-time PCR

	5’ Forward primer 3’
	Reverse primer
*NFKB pathway :*	
AKT1	CTCCTGAGGAGCGGGAGGAGTGG
	GTCCACTCCTCCCGCTCCTCAGGA
MAPK	TATGGCTCTGTGTGTGCTGCTTTTG
	CAAAAGCAGCACACACAGAGCCAT
NFKB	ACAACTATGAGGTCTCTGGGGGTA
	GTACCCCCAGAGACCTCATAGTTGT
*Cytokines production :*	
TNFA	CCATGTTGTAGCAAACCCTCAAGCT
	AGCTTGAGGGTTTGCTACAACATGG
IL1Β	CAGATGAAGTGCTCCTTCCAGGACC
	GGTCCTGGAAGGAGCACTTCATCTG
MCP1	CGCTCAGCCAGATGCAATCAATGCC
	GGCATTGATTGCATCTGGCTGAGCG
*Fatty acids metabolism :*	
ALOX5	GGCATTGATTGCATCTGGCTGAGCG
	CGCTCAGCCAGATGCAATCAATGCC
PTGS2	CTGGGCCATGGGGTGGACTTAAATC
	GATTTAAGTCCACCCCATGGCCCAG
*Oxydation system :*	
MGST1	TTGACAAGAAAGGTTTTTGCCAATC
	GATTGGCAAAAACCTTTCTTGTCAA
NOS2	CCATGGAACATCCCAAATACGAGT
	CACTCGTATTTGGGATGTTCCATG
HO-1	AAGATTGCCCAGAAAGCCCTGGAC
	AACTGTCGCCACCAGAAAGCTGAG
*Housekeeping genes :*	
RPL37A	
	GGTGCCTGGACGTACAATACCACTT
	AAGTGGTATTGTACGTCCAGGCACC
18S	CCATTGGAGGGCAAGTCTGGTGCC
	TGGCACCAGACTTGCCCTCCAATGG

### Statistical analyses

Gene expression levels were analyzed using SAS software (version 9.12). Experimental results are reported as means ± S.E. from at least three independent experiences performed each in triplicate. Treatments were compared to control and to different concentrations using the least square means. The level of significance was defined as *P* < 0.05 for all tests.

## Results

### Effects of n-3 FAs on cytokine gene expression

**Fig. 1:**
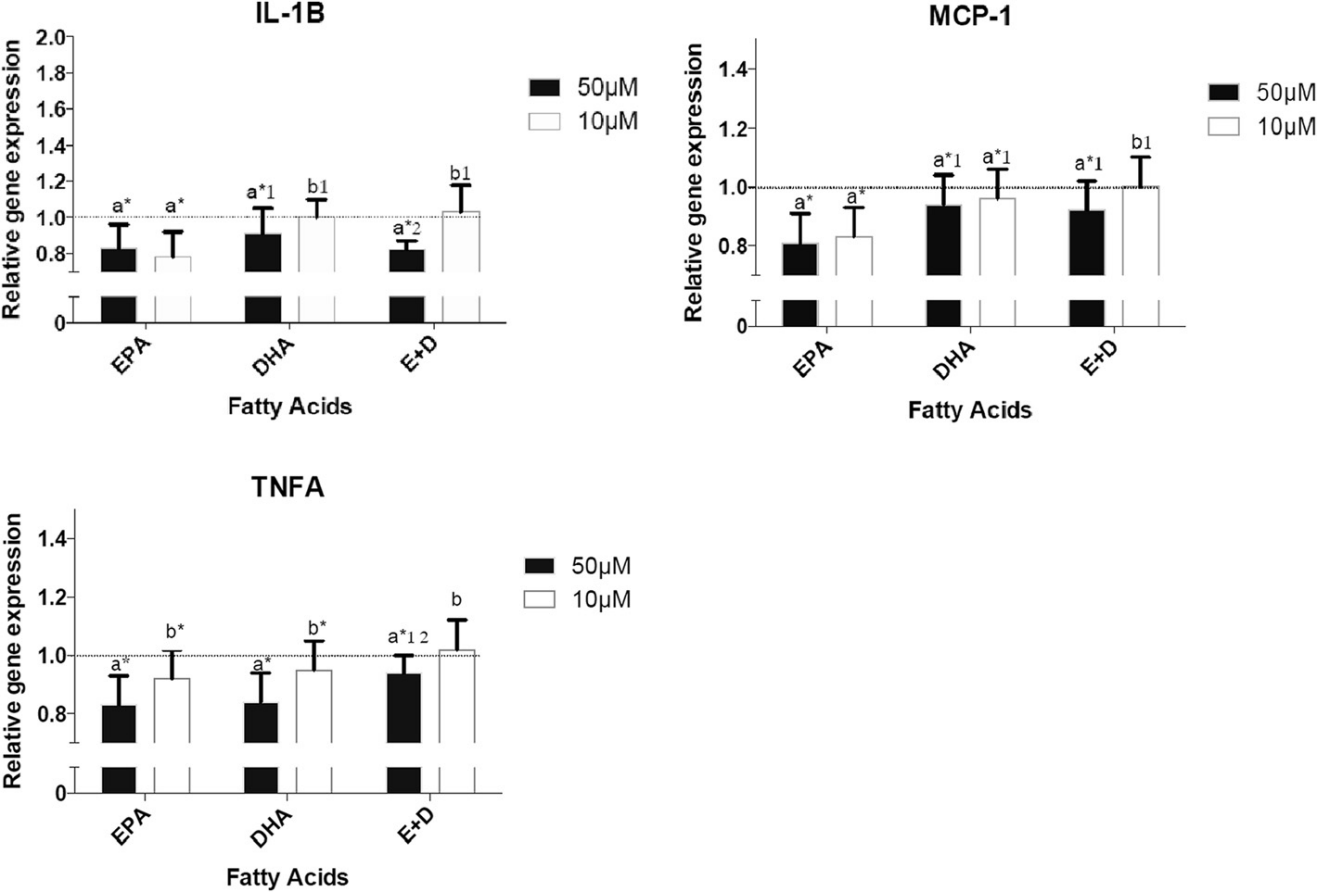
Effects of n-3 FAs on cytokine gene expression after a 24 h incubation of THP-1 macrophages in 10 μM and 50 μM of EPA, DHA and EPA + DHA. ^a,b^ Represents the differences (*P* ≤ 0.05) between the 2 concentrations for each omega-3 FAs and for the mixture EPA + DHA. *P ≤ 0.05 relative to DMSO; ^1^ *P* ≤0.05 relative to EPA within the same concentration; ^2^ *P* ≤ 0.05 relative to DHA within the same concentration

EPA significantly reduced the expression of genes coding for cytokines *IL1B*, *MCP1* and *TNFA,* Fig. 1. EPA concentrations of 10 and 50 μM equally reduced the expression of *MCP1* and *IL1B*. As far as *TNFA* was concerned 50 μM of omega-3 FA reduced its expression more effectively than 10 μM. When cells were incubated with 50 μM of DHA, the down-regulation of *TNFA* and *IL1B* was more intense than with10 μM. The expression of *MCP1* was equally decreased with 50 or 10 μM of both EPA and DHA. The mixture EPA + DHA down regulated the expression of *MCP1, TNFA* and *IL1B* but only with the concentration of 50 μM while 10 μM of EPA + DHA did not affect their expression. Except for *TNFA* on which EPA and DHA had the same effect; EPA seemed to have a more powerful action on the expression of *MCP1* and *IL1B* than DHA and EPA + DHA.

### Effects of n-3 FAs on the expression of genes involved in FA

**Fig. 2:**
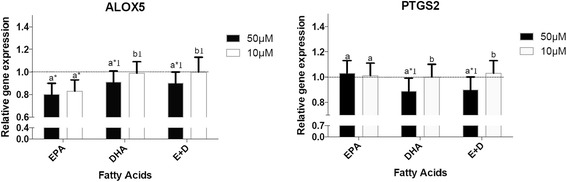
Effects of n-3 FAs on the expression of genes involved in FA metabolism. ^a,b^ Represents the differences (*P* ≤ 0.05) between the 2 concentrations for each omega-3 FAs and for the mixture EPA + DHA. *P ≤ 0.05 relative to DMSO; ^1^ *P* ≤0.05 relative to EPA within the same concentration; ^2^ *P* ≤ 0.05 relative to DHA within the same concentration

The effect of EPA, DHA and EPA + DHA on the expression of two genes involved in the FA metabolism (*ALOX5* and *PTGS2)* is presented in Fig. 2. Relative to vehicle control-related cells, 10 and 50 μM of EPA equally decreased the expression of *ALOX5*. DHA and EPA + DHA also decreased its expression, but only when the concentration of 50 μM was used. The expression of *PTGS2* was also modulated by the omega-3 FAs. A treatment of THP-1 cells with 50 μM of DHA and EPA + DHA reduced its expression when EPA did not have any effect on *PTGS2* gene expression levels.

### Effects of n-3 FAs on NF-κB pathway gene expression

**Fig. 3:**
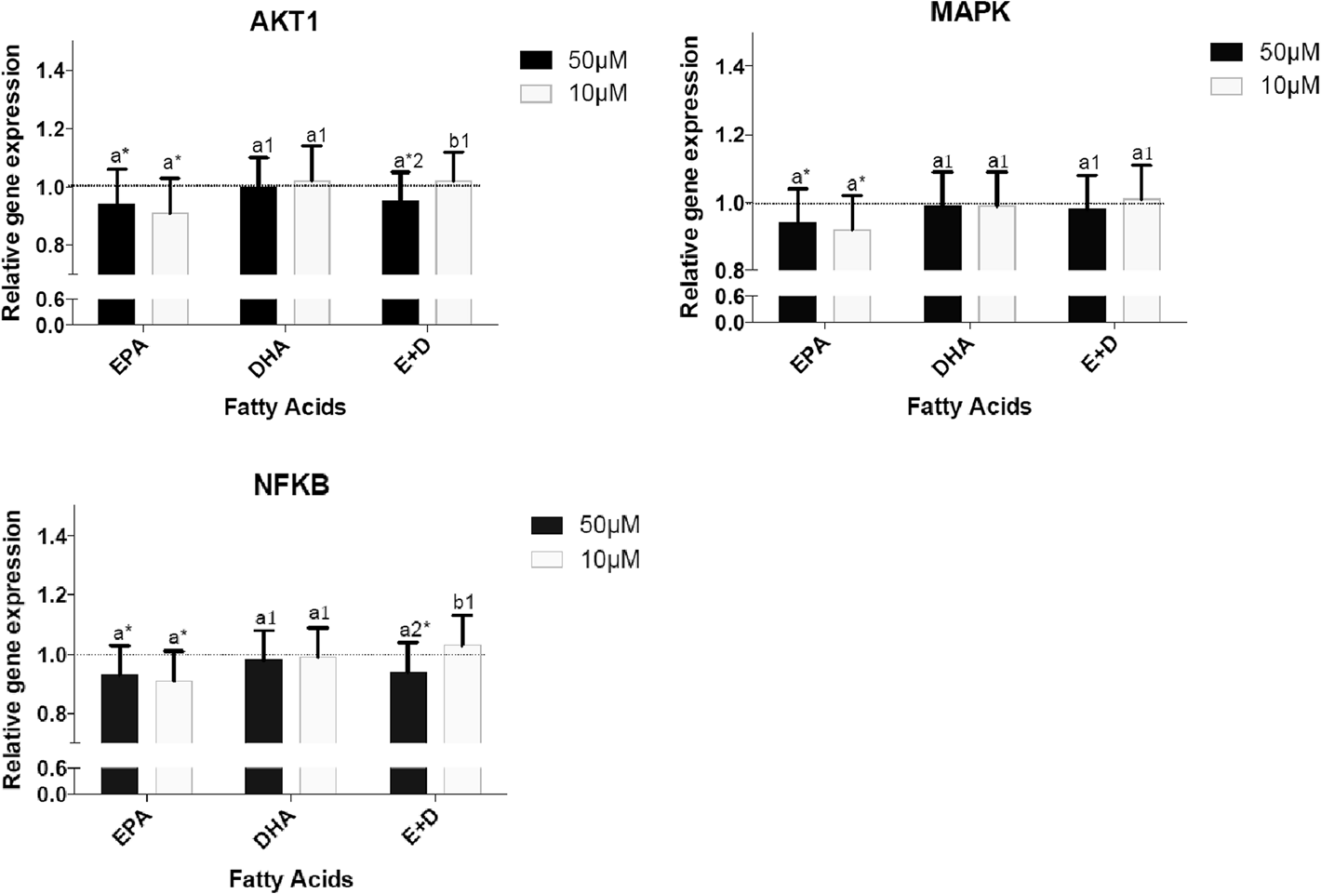
Effects of omega-3 FAs on the expression of genes involved in NF-κB pathway. ^a,b^ Represents the differences (*P* ≤ 0.05) between the 2 concentrations for each omega-3 FAs and for the mixture EPA + DHA. *P ≤ 0.05 relative to DMSO; ^1^ *P* ≤0.05 relative to EPA within the same concentration; ^2^ *P* ≤ 0.05 relative to DHA within the same concentration

Figure 3 shows the effect of EPA, DHA and EPA + DHA on three genes (*AKT1, MAPK and NFKB)* involved in the *NFKB* pathway. 50 and 10 μM of EPA equally inhibited the expression of each of these genes. No significant changes in the expression of *NFKB, MAPK* and *AKT1* were seen when cells were incubated in DHA. The mixture EPA + DHA at a concentration of 50 μM decreased the expression of *NFKB* and *AKT1* but had no effect on *MAPK*. The concentration of 10 μM did not change the expression of these 3 genes. EPA seemed to be more effective on inhibiting *NFKB, MAPK and AKT1* than DHA or the mixture EPA + DHA.

### Effects of n-3 FAs on the expression of oxidative stress genes

**Fig. 4:**
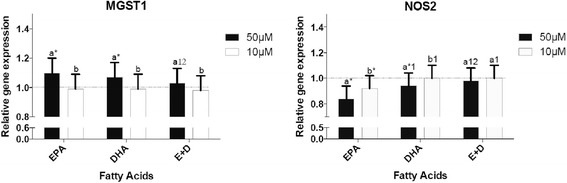
Effects of n-3 FAs on the expression of genes involved in oxidative stress. ^a,b^ Represents the differences (*P* ≤ 0.05) between the 2 concentrations for each omega-3 FAs and for the mixture EPA + DHA. *P ≤ 0.05 relative to DMSO; ^1^ *P* ≤0.05 relative to EPA within the same concentration; ^2^ *P* ≤ 0.05 relative to DHA within the same concentration

Finally, we studied changes in the expression of two genes involved in the oxidative stress (*MGST1* and *NOS2*-Fig. 4). At a concentration of 50 μM EPA and DHA up-regulated the expression of *MGST1* whereas 10 μM did not have any effect. For *NOS2,* 50 μM of EPA and DHA decreased its expression more efficiently than 10 μM. The mixture EPA + DHA did not have any effect on the expression of these genes. EPA had a more powerful effect on the expression of *MGST1* and *NOS2* than DHA or the mixture EPA + DHA.

## Discussion

Inflammation is a condition which contributes to a range of human diseases [7, 27]. EPA and DHA are the major n-3 FAs found in oily fish and fish oil supplements. There is substantial evidence that these FAs are able to partly inhibit several aspects of inflammation, including production of inflammatory cells [27] and eicosanoids from n-6 [31]. The literature had shown that the influence of EPA and DHA on immune cell functions is mediated by several mechanisms [4]. In this study, we focused on the action of EPA and DHA on the regulation of inflammatory gene expression levels on unstimulated THP-1 cells. Literature showed that an increased intake of EPA and DHA resulted in an increased incorporation of n-3 FAs in membrane phospholipids of immune cells [6]. N-3 FAs can then modulate the expression of several inflammatory genes by interacting with various nuclear receptor and transcription factors [4]. NFKB is a key transcription factor involved in the up-regulation of cyclooxygenase (*COX2)* gene, adhesion molecules and inflammatory cytokines [14, 26]. Previous studies reported that fish oil decrease LPS-induced activation of NFKB in human monocytes [17, 32]. Singer et al.*,* suggested that EPA and DHA could inhibit NFKB activity at several levels during NF-κB pathway [32]. Following this lead, we studied in unstimulated macrophages, the expression levels of genes involved in this pathway. We demonstrate that EPA and EPA + DHA, but not DHA, slightly depressed the expression of genes involved in NF-κB pathway (*AKT1*, *MAPK* and *NFKB*). The modulation of these genes expression being very low, we can hypothesise that in unstimulated macrophages the action of EPA and DHA does not seem to go through NF-κB pathway alone. Other mechanisms might be involved.

In addition, EPA suppressed the mRNA expression of pro-inflammatory cytokines (*IL1B, MCP1* and *TNFA*) in THP-1 monocyte-derived macrophages. As mentioned previously, in inflammatory conditions, NFKB plays key roles in up-regulating pro-inflammatory cytokine gene transcription [9]. The results of this study suggest that the effects observed on cytokines can reflect upstream actions on genes from the NF-κB pathway. Effects on gene expression being relatively modest, we can also hypothesise that n-3 FAs may act on expression levels of cytokine genes by other mechanisms.

It is important to highlight the fact that the aim of the present study was to investigate the effect of EPA, DHA and EPA + DHA on the expression of inflammatory genes, in cells under baseline inflammatory conditions. Studies investigating the effects of n-3 FAs on inflammation without induction of inflammation are limited. In most in vitro studies, induction of inflammation with different chemicals (LPS, TNFA, and interferon gamma) is triggered to evaluate the effects of n-3 FAs [19]. Changes in gene expression levels are thus more important in these models. It might be a reason why the effects of n-3 FAs are not as pronounced here as in studies with induced inflammation. In vitro and clinical [2] studies showed that EPA, DHA and fish oil supplements can decrease production of pro-inflammatory cytokines. In addition, a study by Wang and coll., on macrophages treated with EPA and DHA showed reduced expression of *TNFA, IL6* and *MCP1* compared to controls [30]. These results highlight the ability of EPA, DHA and EPA + DHA to down-regulate pro-inflammatory markers, even in the absence of pro-inflammatory stimuli thus suggesting a protective effect of n-3 FAs on THP-1 macrophages inflammatory profile concordant with reported health benefits (7). Combined with data from the present study, these findings suggest that the inverse relationship between n-3 FAs and inflammation may be partly due to their ability to modulate macrophage cytokine secretion via NF-κB pathway.

Eicosanoids are biologically active lipid mediators produced from PUFA, mainly AA. They are important mediators of inflammation [16]. The prostaglandin-endoperoxide synthase (PTGS; also called cyclooxygenase (COX)) and the lipoxygenase (ALOX) are two enzymes contributing to the synthesis of eicosanoids. AA is abundant in membrane phospholipids. It produces pro-inflammatory eicosanoids through these enzymes [5]. EPA and DHA are weaker substrates for ALOX5 and PTGS2 than AA. They produce less potent eicosanoids and lipid mediators (resolvins, maresins, protectins), with an anti-inflammatory profile. The present study shows a dose and n-3 FAs dependent reduction of the expression of *ALOX5* and *PTGS2* when cells are exposed to these FAs. As shown in the literature, the incorporation of EPA and DHA into inflammatory cells occurs at the expense of AA [6]. This might explain the decrease in gene expression levels of *PTGS2* and *ALOX5*.

We also studied the effect of n-3 FAs on the expression of genes involved in oxidative stress. Reactive oxygen species (ROS) are produced by the body and are important in normal cellular functioning. Neutralising ectopic oxidative stress is very important to maintain the body’s integrity. The potential role of oxidative stress as an early event in inflammatory diseases has been widely noted [23]. To prevent abnormal oxidative stress, several physiological anti-oxidant systems act as protective mechanisms. Microsomal glutathione S- transferase 1 (MGST1) and heme oxygenase (HO-1) are enzymes which detoxify reactive intermediates. Nitric Oxide Synthase (NOS) catalyzes the production of nitric oxide (NO), a free radical, as a defense mechanism. During pathological inflammation NO is produced in an excessive manner leading to cell damages. Figure 4 suggests that even when inflammation is not induced, n-3 FAs act on oxidative stress by reducing the expression of a pro-oxidative gene (*NOS2*) and enhancing the expression of *MGST1*, an anti-oxidative gene. In the previous study led by our laboratory [24], the 30 participants had a body mass index between 25 and 40 kg/m^2^. It means that they were overweight to obese participants. Thus the results of this study were from individuals with a potential chronic inflammatory state whereas our in vitro study was performed on unstimulated macrophages. In the clinical trial, each participant received 5 capsules containing 1 g oil each per day (1.9 g EPA and 1.1 g DHA). It is of note that the responses observed in this human clinical study may be modulated by genetic variations that influenced the individual response to n-3 FAs supplementation. These facts might be partly explaining the difference regarding MGST1 expression between the human and the in vitro studies. It is also important to consider the fact that the in vitro study considerably simplifies what is observed in the human body. In fact it doesn’t really take into account different interactions that can be observed in the human body. The role of n-3 FAs on the restoration of free radical homeostasis is not fully understood although studies suggest that EPA and DHA reduce oxidative damages in human and animals [1, 18].

N-3 FAs have beneficial effects on inflammation [4]. It is important to know whether effects of each o n-3 FAs on inflammation are similar. This study shows that in unstimulated macrophages, depending on the metabolic pathway of interest, the n-3 FA having the most important effect is different. Globally, EPA has the most potent effect on gene expression comparatively to DHA and EPA + DHA. Interestingly, in most studies on the effect of EPA and DHA on inflammatory genes, DHA was a more potent inhibitor of inflammatory genes than EPA [11, 12, 19, 21]. However, inflammation was induced in these studies while in the present study effects of n-3 FAs on macrophages were studied without induced inflammation. The greater effect of DHA seems to come from its secretion of more anti-inflammatory lipid mediators (Resolvin D, maresins, protectins) than EPA (Resolvin E) [6, 25]. It could explain that in studies where inflammation is induced, the effects of DHA on expression levels of inflammatory genes were more intense than EPA. Indeed, the induction of inflammation triggers the synthesis of resolvins and other lipid mediators derived from n-3 FAs not seen in cells when inflammation is not induced. Stimulation of a macrophage cell culture with an inflammatory agent may lead to different results but this need to be confirmed.

A dose effect of the omega-3 FA is also seen. Globally incubating the cells with 50 μM of each omega-3 FAs seems to be more efficient then with 10 μM. Thus, when studying the effects of omega-3 FAs, the concentrations must be carefully set in order to truly observe their action.

## Conclusion

N-3 FAs may modulate the expression of genes related to inflammation. In fact these FAs seem to down-regulate the expression of genes promoting inflammation (cytokines, NF-κB pathway) and up-regulate the expression of *MGST1* involved in the prevention of inflammation through detoxification of ROS. The n-3 FAs used in this study have distinct effects on gene expression, EPA having a more potent effect than DHA and or EPA + DHA combined. A dose effect was also seen, in fact higher concentrations (50 μM) being more potent than lower ones (10 μM).
